# Supplementary material for: High-throughput iNaturalist image analysis reveals flower color divergence in Monarda fistulosa
Source: bioRxiv. 2025 May 26:2025.05.21.655392. Preprint. [Version 1] doi: 10.1101/2025.05.21.655392 (PMC12154663; doi:10.1101/2025.05.21.655392)
Supplement: Supplement 1 [file NIHPP2025.05.21.655392v1-supplement-1.pdf]

bioRxiv preprint doi: https://doi.org/10.1101/2025.05.21.655392; this version posted May 26, 2025. The copyright holder for this preprint (which was not certified by peer review) is the author/funder, who has granted bioRxiv a license to display the preprint in perpetuity. It is made available under aCC-BY 4.0 International license.

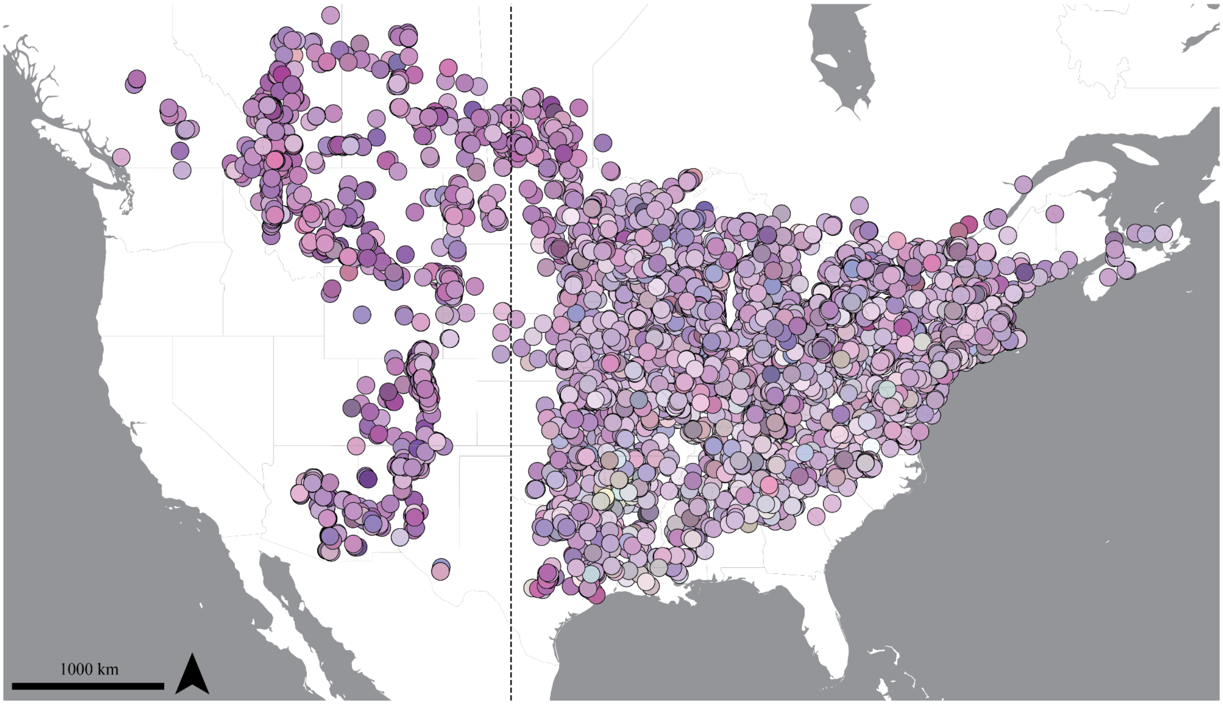

Figure S1: All individual iNaturalist observations mapped using their inferred color. These individual observations underlie the grid-averaged result in Figure 2A. The dotted line indicates -100° longitude.

bioRxiv preprint doi: https://doi.org/10.1101/2025.05.21.655392; this version posted May 26, 2025. The copyright holder for this preprint (which was not certified by peer review) is the author/funder, who has granted bioRxiv a license to display the preprint in perpetuity. It is made available under aCC-BY 4.0 International license.

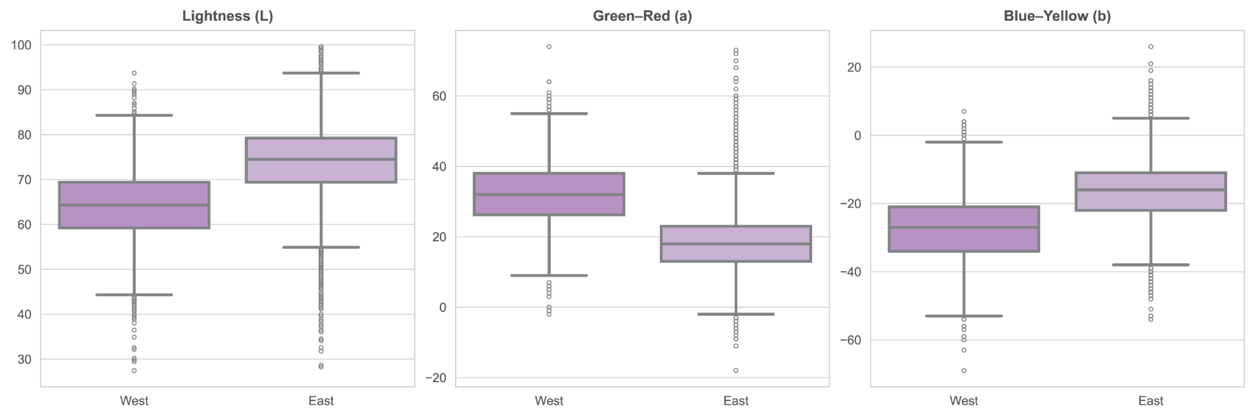

Figure S2: Boxplots summarizing CIELAB color components west and east of -100° longitude, with each box showing the median and interquartile range, and with the color of each box reflecting the geometric median CIELAB value from west and east, respectively.

bioRxiv preprint doi: https://doi.org/10.1101/2025.05.21.655392; this version posted May 26, 2025. The copyright holder for this preprint (which was not certified by peer review) is the author/funder, who has granted bioRxiv a license to display the preprint in perpetuity. It is made available under aCC-BY 4.0 International license.

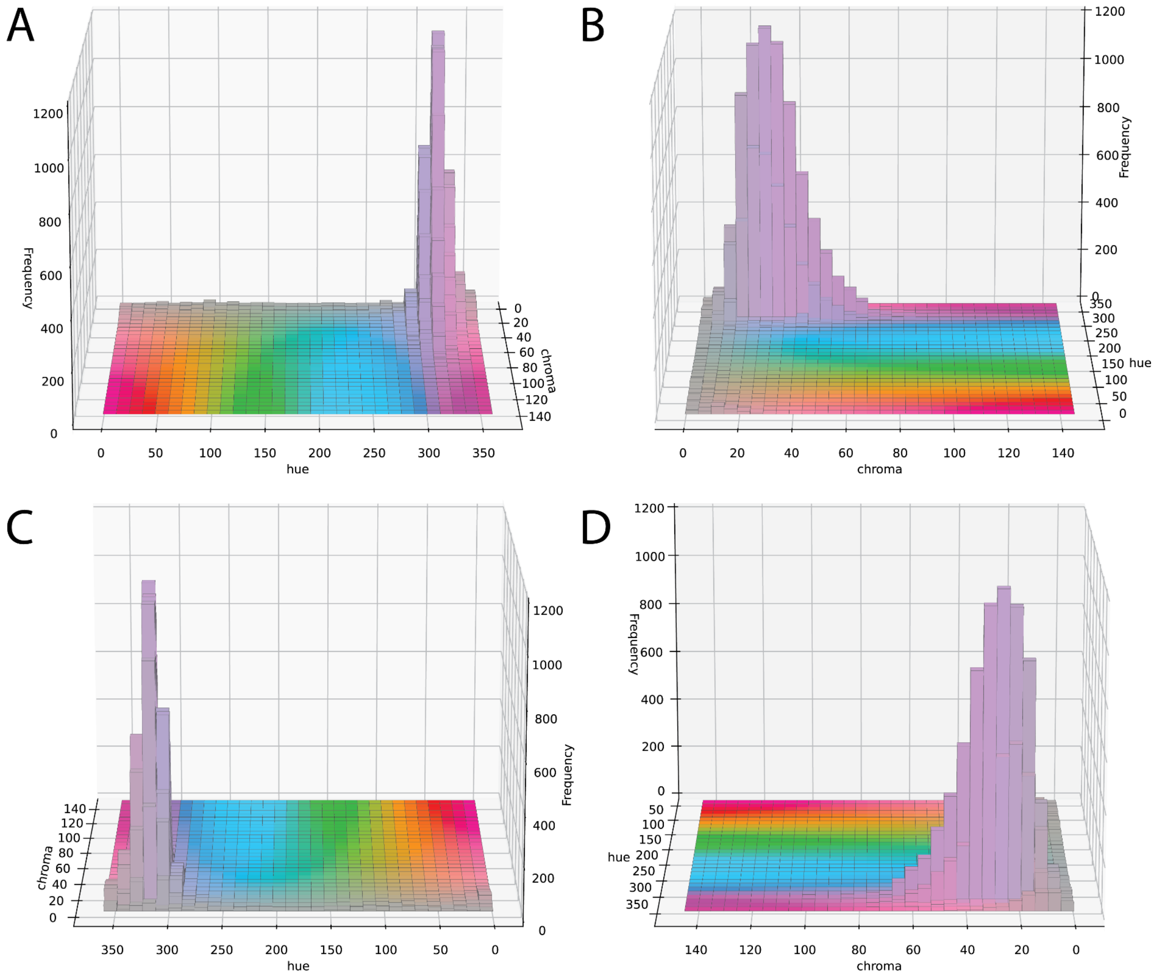

Figure S3: Complete rotation (A-D: 0°,90°,180°,270°) of the three-dimensional histogram from Figure 3A, showing frequency of observations across values of chroma and hue from LCh color space (lightness held constant at value 70).

bioRxiv preprint doi: https://doi.org/10.1101/2025.05.21.655392; this version posted May 26, 2025. The copyright holder for this preprint (which was not certified by peer review) is the author/funder, who has granted bioRxiv a license to display the preprint in perpetuity. It is made available under aCC-BY 4.0 International license.

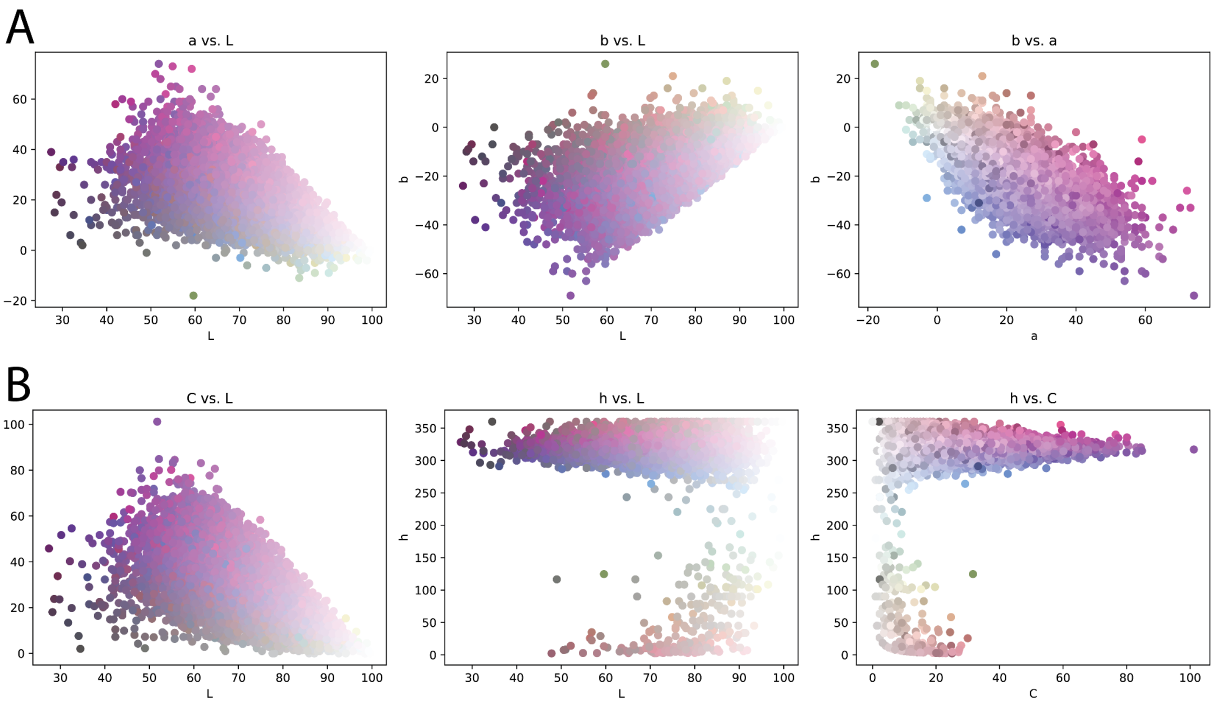

Figure S4: Pairwise projection of color components in A) CIELAB color space, and B) LCh color space. The bottom-right panel is identical to Figure 3B.

bioRxiv preprint doi: https://doi.org/10.1101/2025.05.21.655392; this version posted May 26, 2025. The copyright holder for this preprint (which was not certified by peer review) is the author/funder, who has granted bioRxiv a license to display the preprint in perpetuity. It is made available under aCC-BY 4.0 International license.

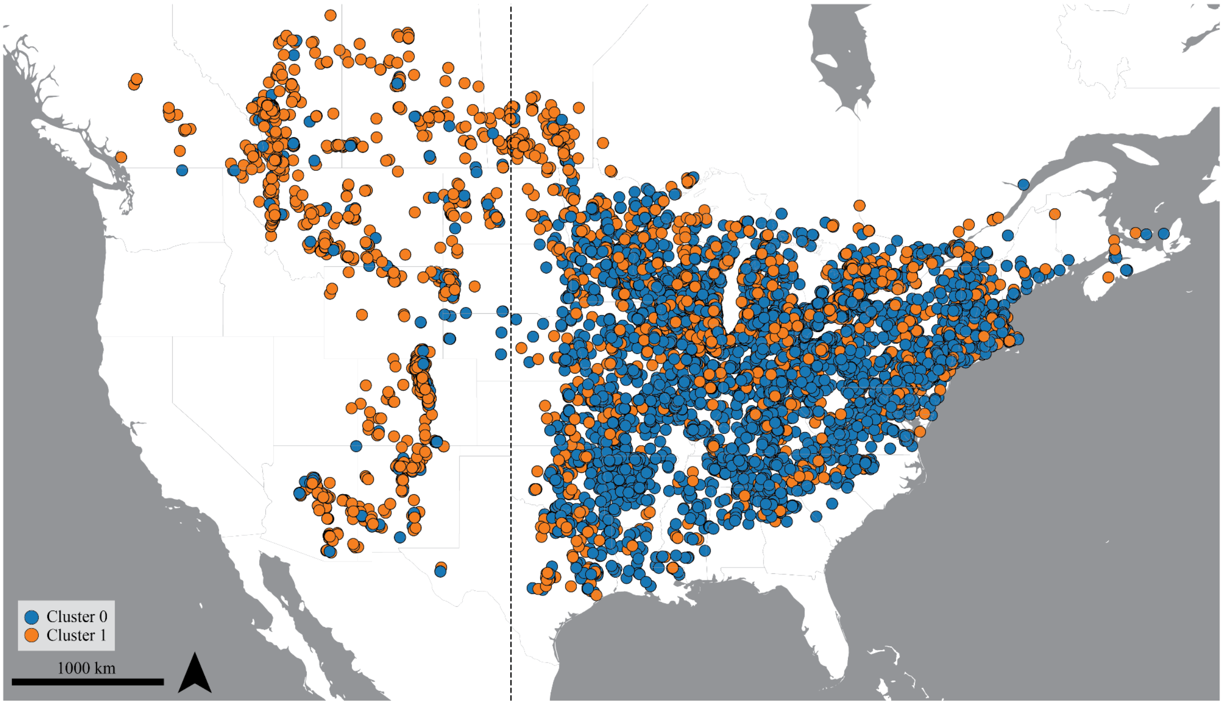

Figure S5: K-means clustering on the CIELAB color components with k=2. Color of point represents cluster identity for each observation. Sliding window averaging from this data across longitudes produced Figure 3D. The dotted line indicates -100° longitude.
